# Cyclic growth of hierarchical structures in the aluminum-silicate system

**DOI:** 10.1186/s13322-015-0007-9

**Published:** 2015-03-06

**Authors:** Agnieszka Dyonizy, Vitaliy Kaminker, Joanna Wieckowska, Tomasz Krzywicki, Jim Pantaleone, Piotr Nowak, Jerzy Maselko

**Affiliations:** Department of Chemistry, Technical University, Wroclaw, Poland; Department of Chemistry, University of Alaska Anchorage, Anchorage, AK USA; Department of Physics, University of Alaska Anchorage, Anchorage, AK USA

**Keywords:** Hierarchy, Complex systems, Chemical garden, Self-construction

## Abstract

**Background:**

Biological structures grow spontaneously from a seed, using materials supplied by the environment. These structures are hierarchical, with the ‘building blocks’ on each level constructed from those on the lower level. To understand and model the processes that occur on many levels, and later construct them, is a difficult task. However interest in this subject is growing. It is now possible to study the spontaneous growth of hierarchical structures in simple, two component chemical systems.

**Results:**

Aluminum-silicate systems have been observed to grow into structures that are approximately conical. These structures are composed of multiple smaller cones with several hierarchical levels of complexity. On the highest level the system resembles a metropolis, with a horizontal resource distribution network connecting vertical, conical structures. The cones are made from many smaller cones that are connected together forming a whole with unusual behavior. The growth is observed to switch periodically between the vertical and horizontal directions.

**Conclusion:**

A structure grown in a dish is observed to have many similarities to other hierarchical systems such as biological organisms or cities. This system may provide a simple model system to search for universal laws governing the growth of complex hierarchical structures.

**Graphical Abstract:**
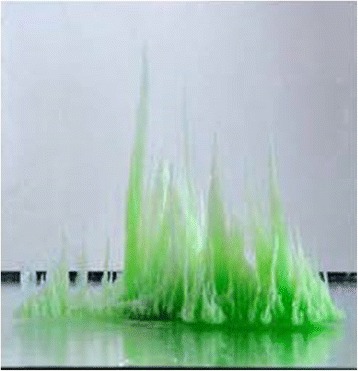
Side view of the chemical structure made from many vertical cones to form a chemical metropolis. The tallest structure is 17 cm high.

## Background

Silicate gardens, known more generally as chemical gardens [1-12], have been known for over 300 years. The structures produced often resemble biological structures such as trees, plants or mushrooms. Also, the processes by which these structures grow qualitatively resemble those in biology: growing spontaneously from a “seed” and constructed from semipermeable membranes that form chemical cells. It has been demonstrated in the laboratory that in these chemical cells, chemicals can diffuse inside, react, and then the products diffuse out [13,14]. Thus chemical gardens may be relevant to the fundamental problem of how life originated [15-17]. More generally, much of the considerable recent interest in chemical gardens follows from the general growth in the study of complex systems, pattern formation and systems chemistry. The structures observed in chemical gardens are often hierarchical and so may be helpful in understanding and mastering the formation of hierarchical structures, which play a large role in biology, artificial biology and technology [18-25]. Interest in chemical gardens is growing and the field was recently given a new name, chemobrionics.

## Results

In the experiments discussed here, aluminum chloride solution is pumped into a container of sodium silicate solution from below. Two different concentrations of chemicals are used, which we denote as Experiment A and Experiment B, see Table 1. Experiment A has a much lower concentration of the aluminum chloride solution than Experiment B. The two experiments are related in that the structure found in A appears as a ‘building block’ of the structure observed in Experiment B.

**Table 1:** **Chemical concentrations and densities used in the two experiments**

	**Interior (pumped) solution**	**Exterior (tank) solution**
Experiment A	0.30 M AlCl_3_	0.30 M sodium silicate
	1.031 g/cm^3^	pH = 10.5
		1.021 g/cm^3^
Experiment B	1.5 M AlCl_3_ plus green food coloring,	0.50 M sodium silicate
	1.155 g/cm^3^	pH = 11.7
		1.036 g/cm^3^

**Figure 1:**
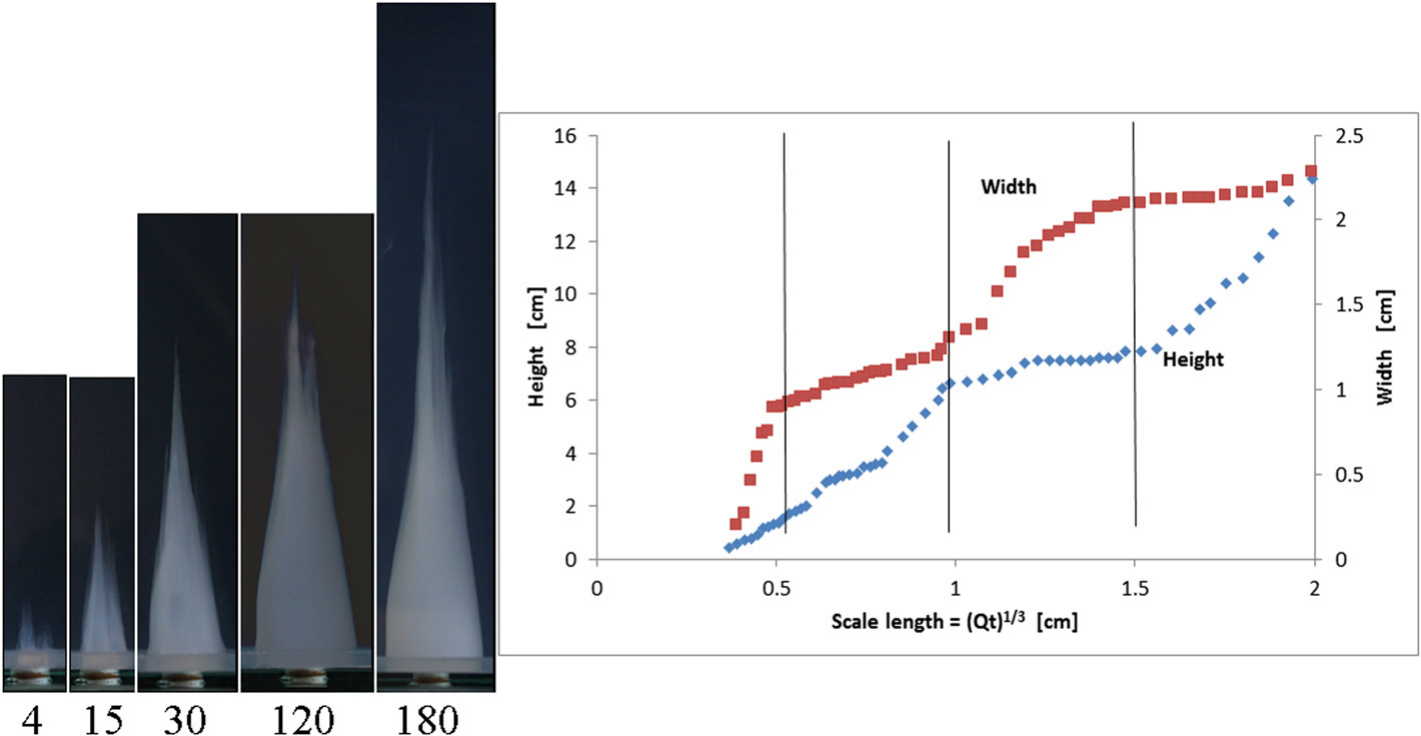
**Growth of conical structures for Experiment A (see Table** **1** **for concentrations). Left**: Photographs of structure at various times since start of experiment, units of time are in minutes (the height of the structures shown are, from left to right, 1.3, 3.6, 6.5, 7.9, and 10.4 cm). **Right**: Height and width of structure as function of volumetric scale length. The vertical lines indicate when growth regimes switch.

For experiment A, a precipitation structure that resembles a cone is formed, see the photographs in Figure 1 (left).

**Figure 2:**
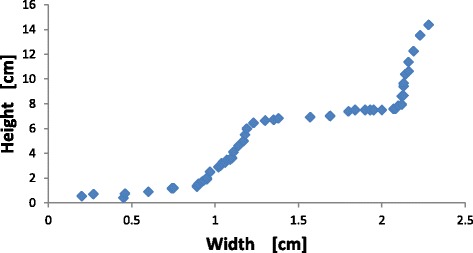
**Plot of height versus width for the structures found in Experiment A.**

In the beginning the density of the metal salt solution is slightly larger than the surrounding silicate solution (see Table 1) so the metal salt solution spreads out along the bottom of the container to a width of about 0.9 cm. As the structure grows the metals salt solution decreases in concentration and density so that it becomes less dense than the surrounding silicate solution causing the growth to change from horizontal to vertical. A few small cones grow vertically and these eventually (at the time of the first vertical line in Figure 1 (right)) merge to form one, single cone. As pumping continues this conical structure grows in both height and width. While the overall structure generally resembles that of a cone, occasionally the vertical growth of the cone bifurcates to form small, separate tips for the cone, however these tips soon merge to form a single cone again. The ratio of the height to the width of the structure is not constant but instead the growth switches between the horizontal and vertical directions, see Figure 1 (right) and Figure 2. In these plots the height is defined as the height of the tallest tower and the width is as measured at the base of the structure in the photo from the side. The switching does not occur at regular intervals in time, but it does occur at somewhat regular intervals in the scale length defined as

1 $$ \mathrm{Scale}\ \mathrm{length}={\mathrm{V}}^{1/3}={\left[\mathrm{Q}*\mathrm{t}\right]}^{1/3} $$

where *V* is the volume of the pumped solution, *Q* = 0.030 mL/sec is the pumping rate and *t* is time. For the experiment shown in Figures 1 and 2, measurements were discontinued after 4 hours.

In classical chemical gardens, tubes with very well defined walls are formed and the tubes are generally empty inside. In this aluminum silicate system, when the cone is cut, the inside of the cone is observed to be approximately a continuous gel. The aluminum chloride solution flows through the gel to construct the conical structure.

**Figure 3:**
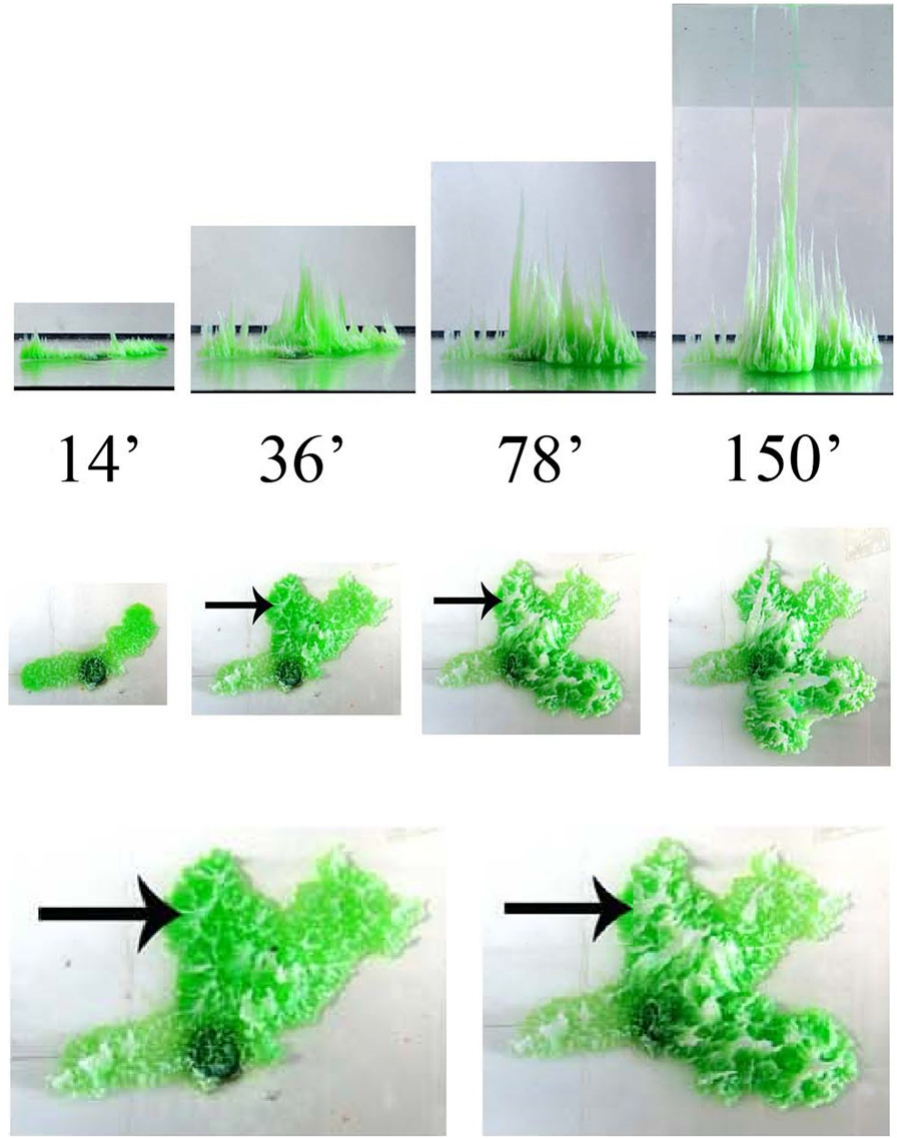
**Photographs of the structure growing in Experiment B.** Green food coloring was added to make the structure more visible. The top two rows show horizontal and vertical views at times 14, 36, 78 and 150 minutes (the height of the tallest tower at these times was 1.0, 6.8, 11.0 and 22.1 cm, respectively). The two pictures on the bottom are magnification of the overhead view at times 36 and 78 minutes. The arrows indicate one particular point on the structure.

For Experiment B, where a much higher concentrations of the interior aluminum chloride solution is used, the structures produced are more complex. Figure 3 shows one such typical structure. Now the pumped aluminum chloride solution has a higher density than the exterior silicate solution (see Table 1) and so it spreads out on the bottom of the experimental container. The horizontal spreading eventually stops due to the formation of precipitate, but as pumping continues the internal pressure increases such that the membrane breaks in random places and horizontal spreading occurs again. The horizontal spreading occurs in such a way as to form many connected, “fingers” along the bottom of the experimental vessel. Inside these horizontal fingers a fluid distribution network forms from which vertical structures grow. Such distribution networks are commonly formed where fluids move through a medium that can be eroded [26]. Presumably the interior aluminum chloride solution is dissolving some of the interior precipitate to create channels inside the horizontal fingers. Such fluid distribution networks are common in biological systems, e.g. arteries in animals or branches in plants [26].

The vertical growth along the horizontal distribution networks takes the form of conical structures similar to those found in Experiment A. The occurrence of vertical growth indicates that the metal salt solution has become dilute, so that buoyant forces can drive the vertical growth. Dilution will occur from osmosis, driving water from the silicate solution into the concentrated metal salt solution, and from depletion due to formation of the precipitate. This dilution probably also explains why the color of the vertical cones is much whiter than that of the precipitate in the base of the structure. There is definitely less of the green dye in the vertical structures. This is consistent with the view that the cones in Experiment B are similar to the ones observed in Experiment A where a less concentrated metal salt solution was used.

**Figure 4:**
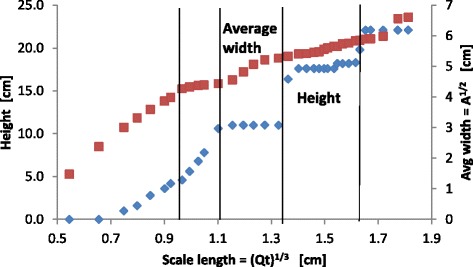
**Maximum height and average width of the structure as function of volumetric scale length for Experiment B.** The vertical lines indicate times when the growth switches between vertical and horizontal.

The vertical growth of the conical structures is not uniform but, as found in Experiment A, often switches to horizontal growth. However for the structure in Experiment B the horizontal growth is not confined to just the cones, instead it can also take the form of new horizontal spreading and finger formation along the bottom of the experimental vessel. To quantify the growth found in Experiment B in a manner similar to that done for Experiment A, we have defined an average width for the entire structure.

2 $$ \mathrm{average}\ \mathrm{width}={A}^{1/2} $$

where *A* is the horizontal area of the structure as viewed from above. The maximum vertical height and average width are plotted versus the scale height in Figure 4 for Experiment B. This figure shows that the growth switches between the vertical and horizontal directions, qualitatively similar to what was found for Experiment A.

## Discussion

A basic building block for vertical growth in both experiments A and B are structures that are approximately cones. While vertically growing conical structures are relatively common, e.g. icicles, stalactites and sandpiles, there are fundamental differences between these structures. For example, icicles, stalactites and sandpiles all grow from flow along the *outside* surface of the structure [27,28] while for the structures observed here the flow is primarily *through* the structure. That there are fundamental difference between how these structures grow is reinforced by the unusual time behavior that is observed for the growth. The growth of the conical structures observed here switches smoothly between the vertical and horizontal directions at roughly equal intervals in the scale length. This is in sharp contrast to icicles and stalactites, which grow smoothly, and to sandpiles which grow via a scale-free distribution of avalanches [28]. These approximately conical structures result from different dynamics but are visually similar.

It is not unusual for different dynamics to lead to common shapes. For example, electrochemical deposition, lightning and viscous fingering involve different physical phenomena but all produce similar patterns and are described by the same mathematical model. Similarly there are complex structures and complex organizations that resemble one another and are described by the same scientific model, for example cells, biological organisms, economies, factories and cities. Such commonalities also occur for the structure found in Experiment B. The horizontal spreading, together with the resource distribution network and the structures that grow vertically from it visually resemble a metropolis. While it is possible that this visual similarity is a coincidence, it seems more probable that the similarity follows from some universal laws that control the growth of structures in these systems.

A metropolis is a very complex, hierarchical system which is shaped by engineering, economics, technology, psychology, sociology, history and geography [29]. All these disciplines appear to be relevant in determining the resultant structure. However most metropolises have a similarity in shape that is rather insensitive to these details. The chemical structures grown in this experiment (Figure 3) differ in size from a metropolis by 6 orders of magnitudes but they have a similar, hierarchical nature.

In hierarchical structures a higher level is made from building blocks on a lower level and this process is repeated on every level. Aluminum in alkaline solution forms different compounds with many levels [30-35]. Very similar and even more complex structures are formed in silicate. The hierarchical levels for our systems of aluminum and silicates in basic solution are the following:

1. Atoms Al, Si, O, H,
2. Simple Al(H_2_O)_6_^3+^, Si(OH)_4_^−^ compounds
3. More complex compound have been detected:
  Al_13_O_4_(OH)_24_(H_2_O)_12_^7+^, Al_2_O_8_Al_28_(OH)_56_(H_2_O)_26_^18+^ and [36,37] Al_2_(OH)_2_(OH_2_)_8_^+4^. Starting from SiO_4_^4−^ centers, the tetrahedrons are joined together forming pairs of (Si_2_O_7_^6−^), ring (Si_6_O_18_^12−^), chains, double chains, sheets, and three-dimensional frameworks [34,38]. In mixtures of aluminum and silicate many compounds exist such as^31^ Si_12_Al_12_O_24_^+12^. The structure and concentration of these compounds depends on the concentrations of initial compounds and the pH. In the solid state one example are such complex compounds are zeolites that are aluminosilicates. At least 206 different zeolite frameworks have been synthesized, however theoretically millions are possible.
4. The compounds mentioned above form permeable gels. Gels may have different compositions depending on concentrations and pH [39].
5. The gels form the membrane, which may have different shapes such as fingers or cones.
6. Cones that have complex interior structure surrounding and containing a permeable membrane.
7. “Cities” made from many cones arranged in lines made from channels that distribute resources.
8. A “metropolis” made from many cities connected together.

For chemical gardens the morphology space of possible structures is surprisingly large. Changes in concentration or composition, of either the inside or outside solutions, can lead to very different structures. Evolution in such nonlinear dynamical systems depends sensitively on the initial components. However here the system is hierarchical, with the components of one level forming the building blocks for the next level. This hierarchical organization appears to restrict the growth trajectories into common paths. Thus the study of these chemical structures may lead to a general theory of hierarchical systems. The chemical structures described here can be grown in a relatively short time compared to the construction of a city and thus may provide a good experimental model for testing theories of structure formation in hierarchical systems.

Besides the visual similarity of the structures in Figure 3 to a metropolis, it is interesting to note that the growth rates are also somewhat similar. The maximum height of a metropolitan area exhibits a staircase shape similar to that seen in Figure 4. The sharp transitions in building heights are related to the use of new materials (e.g. transitions from wood to stone to cement and then to metal), or new construction methods. This is a manifestation of the general observation that, in a complex system, growth is often not a linear process but occurs in jumps. Such staircase growth has been observed previously in complex chemical systems [40,41]. Thus the staircase growth, as seen in Figures 1, 2 and 4, may also be a characteristic related to the hierarchical nature of the system.

In general, jumps in complex systems occur when the concentration of resources reaches a critical point which allows the system to leap forward in its development. This is then followed by a period of relatively low activity until the next jump. For our chemical system the exact mechanism responsible for the staircase growth is not known, however it is probably due to a somewhat similar mechanism. In particular, in traditional chemical gardens periodic growth occurs from pressure relaxation oscillations. Fluid flow into the structure (usually from osmosis) slowly increases the internal pressure, which increases the stress in the thin tube walls until a critical value is reached and membrane rupture occurs which releases the pressure and leads to new tube growth [5-7]. However the structures found in Experiments A and B differ from traditional chemical gardens in that they are not hollow tubes with thin membranes but are relatively continuous permeable gels through which the metal salt percolates outward. The thicker membranes in these structures implies that the stresses are distributed throughout the gel and so ruptures will occur throughout the gel. Internal ruptures will both create channels for fluid flow and redistribute the stresses throughout the structure. These actions will change the nature of the structure growth in ways that are difficult to predict a priori. It seems reasonable to expect that such “thick” membrane pressure oscillations will be qualitatively different than those observed in traditional chemical gardens. We intend to pursue further experiments, measuring the internal pressure of the structures while they grow, to determine if the staircase growth seen in Figures 1, 2 and 4 is an example of “thick” membrane pressure relaxation oscillations.

## Experimental

1.5 liters of sodium silicate solution were poured into a rectangular glass container with size: 10 × 10 × 25 cm. A solution of aluminum chloride was then injected into the sodium silicate solution from below by a peristaltic pump (Gilson Minipuls 3) at a constant flow rate of 0.30 mL/sec. Chemicals were supplied by Sigma-Aldrich. All the experiments were carried out at 20±1C temperature. The pH of the silicate solution was changed by adding HCl. The experiments were carried out until the observed structures reached either the upper surface of the liquid or the horizontal edge of the reaction vessel. The structures were photographed simultaneously from both the side and overhead.

## Conclusion

Hierarchies are a basic property of nature. They appear in chemical structures, in biology, society and civilization. This suggests that the construction of hierarchical structures is a basic process in evolution. The detailed studies and mathematical modeling of dynamic hierarchies are just beginning, therefore finding a simple chemical system where they can be easily studied is important [42-45].

The structures observed in silicate gardens are characterized by networks of physical and chemical processes that are organized in space and time. The growth follows trajectories that may be chemically switched to obtain different structures. The structures grow in complexity with time. Until now the only known systems with continuously increasing complexity were biological systems. However biological systems are incredibly complex, with millions of compounds and processes woven together. Chemical gardens are simpler systems, with some properties similar to biological systems, but they can be studied much more easily. Mastering the growth processes of chemical gardens has the potential to make a tremendous impact on science, technology and the economy. It is sure to transform the frontiers of knowledge. It may help us to understand biological growth, and might even contribute to the formation of another branch of life. It will help us to understand and grow complex hierarchical systems, full of emergences, with continuously increasing complexity.
